# Supplementary material for: Disrupted modular organization of primary sensory brain areas in schizophrenia
Source: Neuroimage Clin. 2018 Mar 1;18:682–93. doi: 10.1016/j.nicl.2018.02.035 (PMC5987872; doi:10.1016/j.nicl.2018.02.035)
Supplement: Supplementary file 1 — Supplementary material [file mmc1.docx]

# Supplemental Information

## Overlap of modules between the two populations

We calculated the overlaps between modules in the optimal partitions for the two groups, shown in Fig.S1. The lines and the columns of the matrix correspond to communities in the control and in the patients’ group, respectively, ordered by size (with 1 indicating the largest community). The colored elements of the matrix indicate overlaps between communities in the two groups, and the number of common nodes.

From this figure, it is apparent that schizophrenia subjects show a reorganization of modular structure beyond a simple fragmentation of the larger communities. By way of example, Com1 of the control group (first line) shows significant overlap with 3 communities of the SCZ group (1, 9,and 26). However, community 9 of the patients’ group also shares a substantial number of nodes with community 9 and 13 of the control group, indicating changes in membership that involve rearrangement of multiple modules.


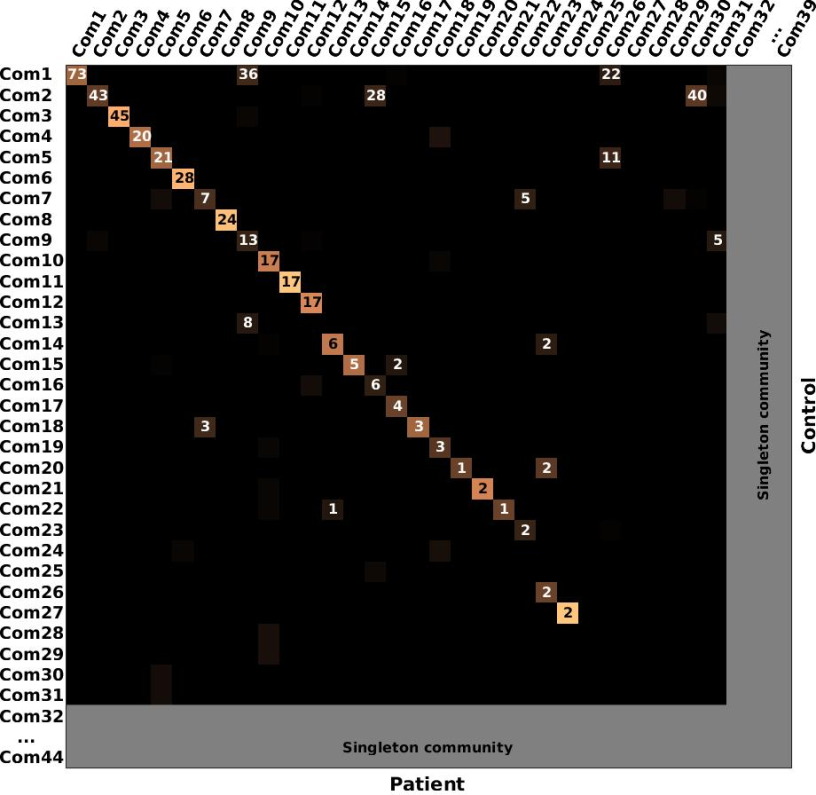


Figure S1. Overlap between the two partitions. The colors represent the percentage of overlap, and the value: the number of nodes in common.

## Connector hubs

Network nodes characterized by simultaneously high degree and high participation coefficient represent the integrative hubs of connectivity networks, and are dubbed "connector hubs". Differences in centrality and connectivity structure between the two populations may result in a different distribution of connector hubs in the brain. Fig.S11 shows the regions with simultaneously high values of degree (degree coefficient*>* 0*.*6) and participation coefficient (participation coefficient*>* 1) in the SCZ and healthy control groups. Notable differences are observed in the parietal regions, where the superior parietal lobule represents a connector hub in control subjects, but not in patients. Importantly, the Broca area appears as prominent connector hub only in the SCZ subjects.

| 1. **Control** |
| --- |
| 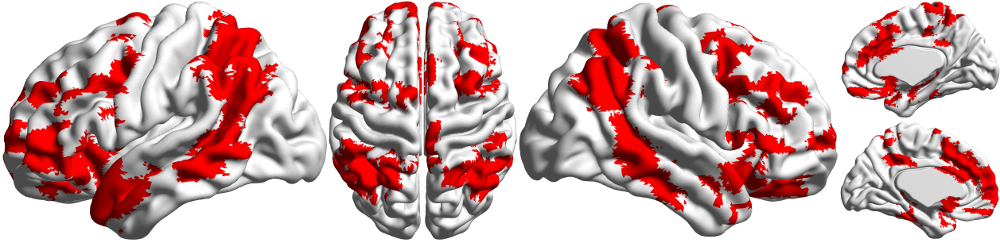 |
| 1. **Patient** |
| 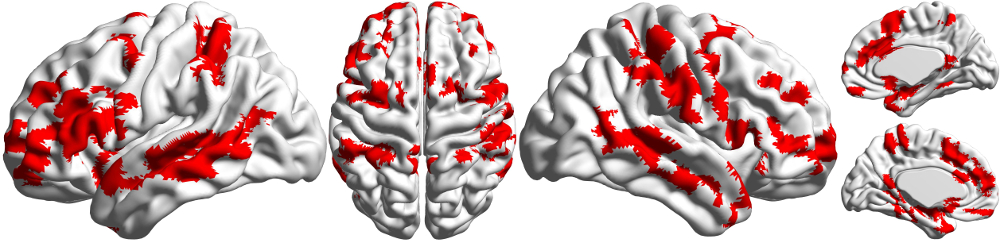 |

Figure S2. Anatomical distribution of connector hubs, defined as nodes with participation coefficient higher than 0.6 and degree higher than 1.

## Effect of Age

| 1. **Up to 25 years old (21 subjects)** |
| --- |
| 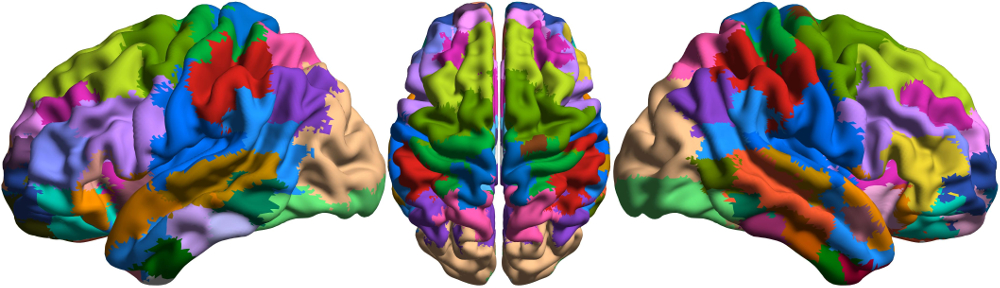 |
| 1. **Up to 35 years (39 subjects)** |
| 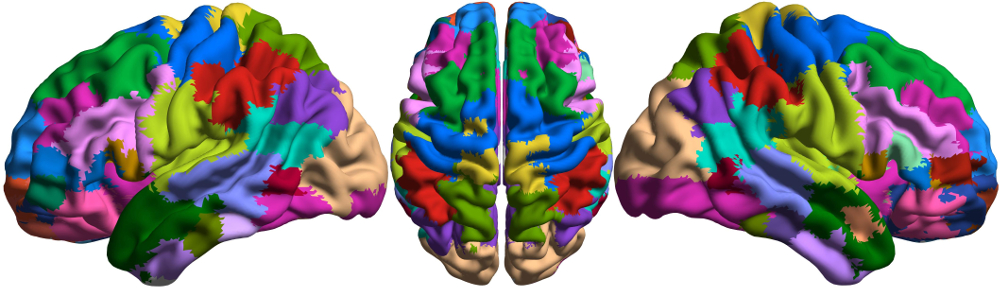 |
| 1. **More than 35 years old (39 subjects)** |
| 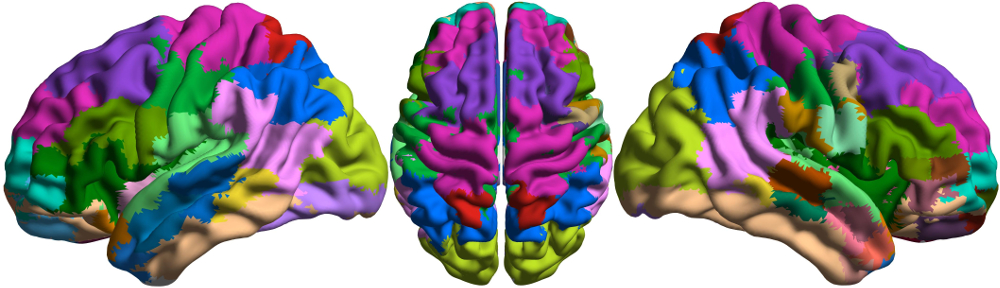 |

Figure S3. Maps representation of the communities for different age groups. A. Patients from 18 to 25 years old; B. Patients from 18 to 35 years old; C. Patients from 36 to 65 years old.

## Effects of pharmacological treatment

| 1. **Same year of first treatment (18 subjects)** |
| --- |
| 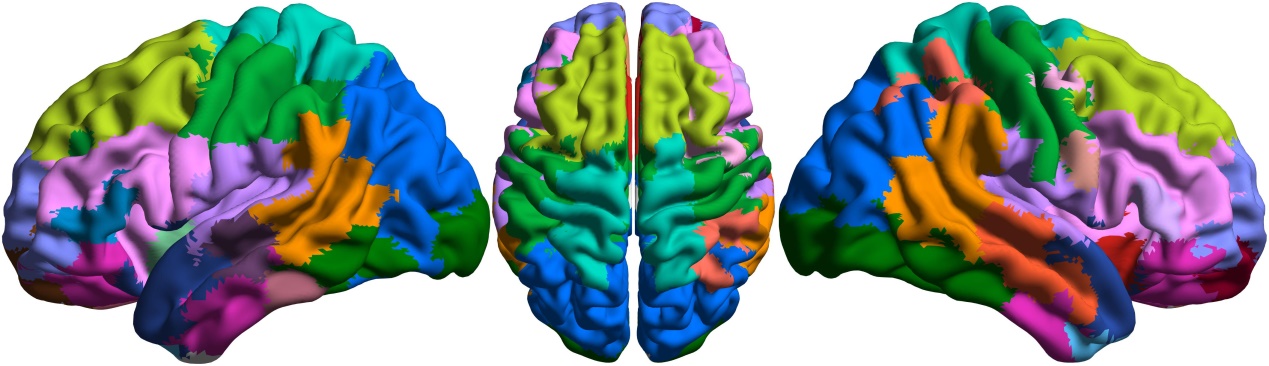 |
| 1. **Within 1 year of first treatment (35 subjects)** |
| 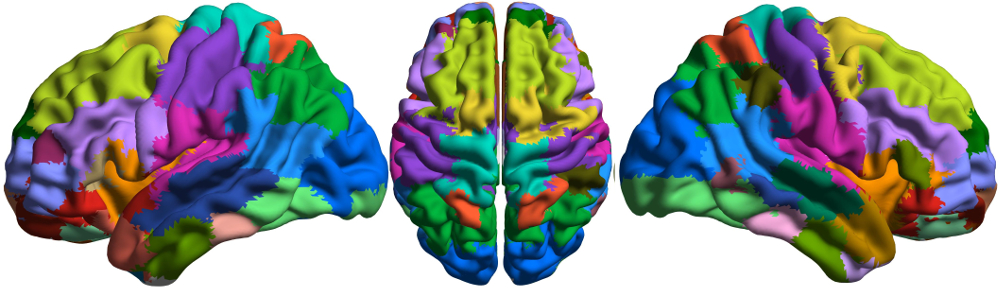 |
| 1. **After 5 years or more of treatment (16 subjects)** |
| 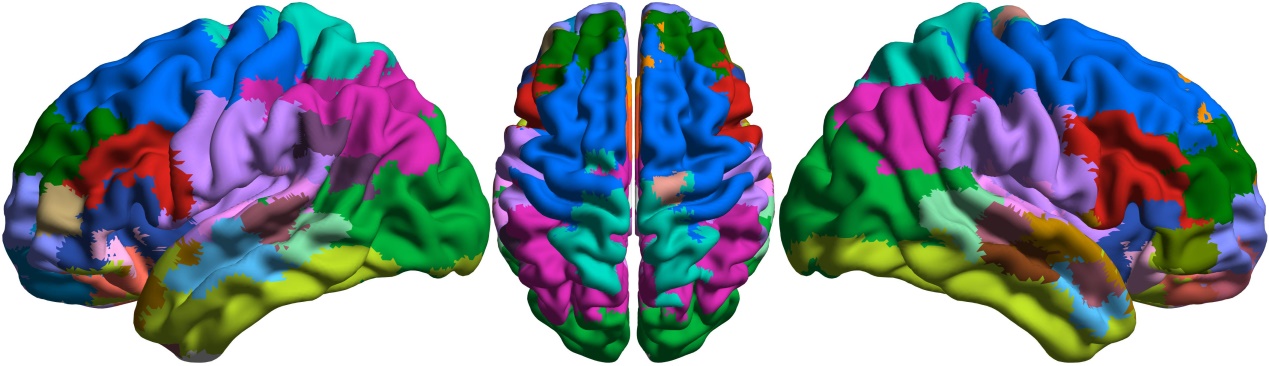 |

Figure S4. Maps representation of the communities for different pharmacologicaltreatment history groups. A. Patients scanned the same year than the first treatment; B. Patients scanned in the year of the first treatment ; C. Patients scanned more than 5 years after the first treatment.

**Effects of motion: motion correction**

**A. Control group**

**
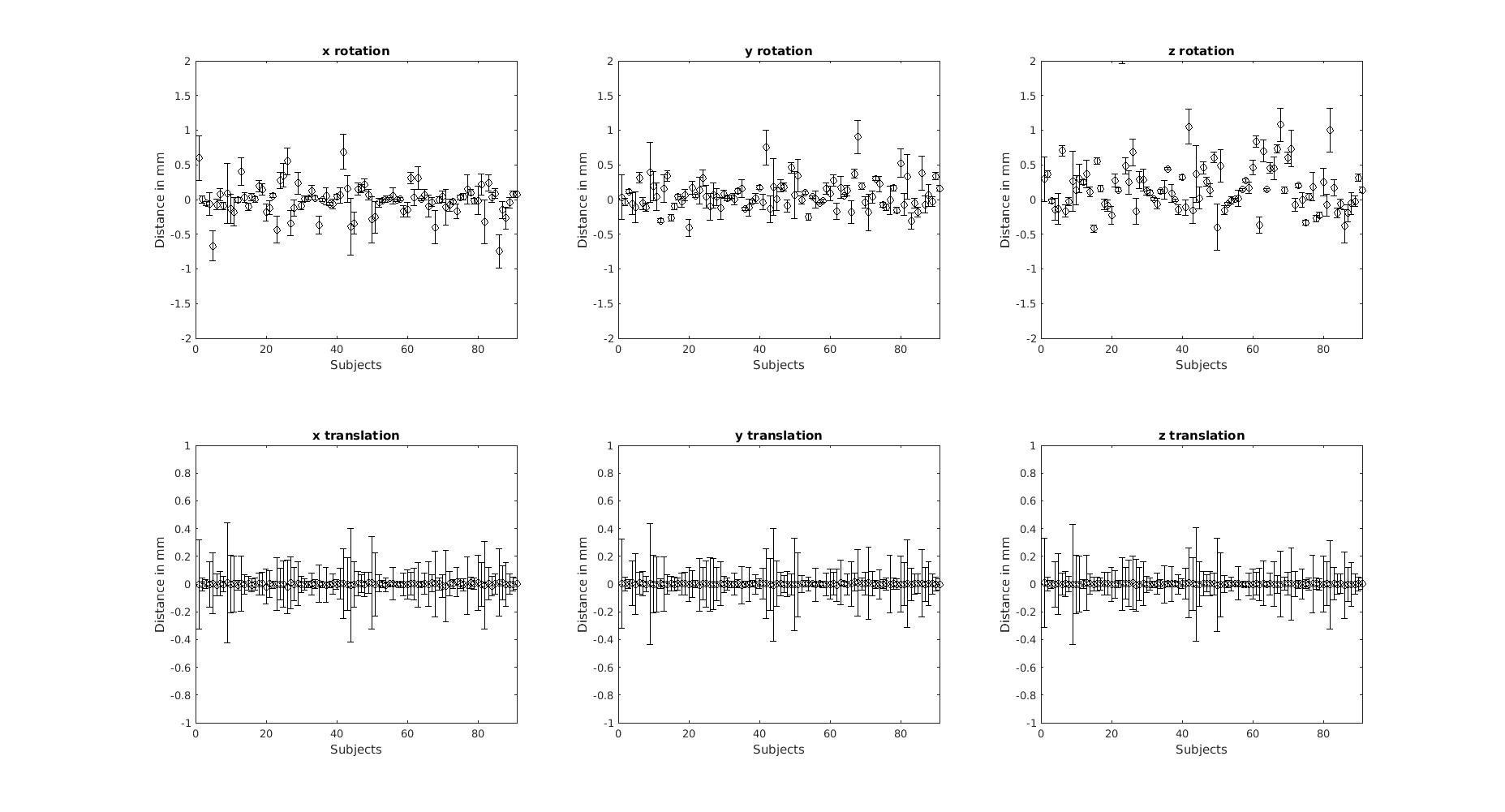
**

**B. Patients’ group**

**
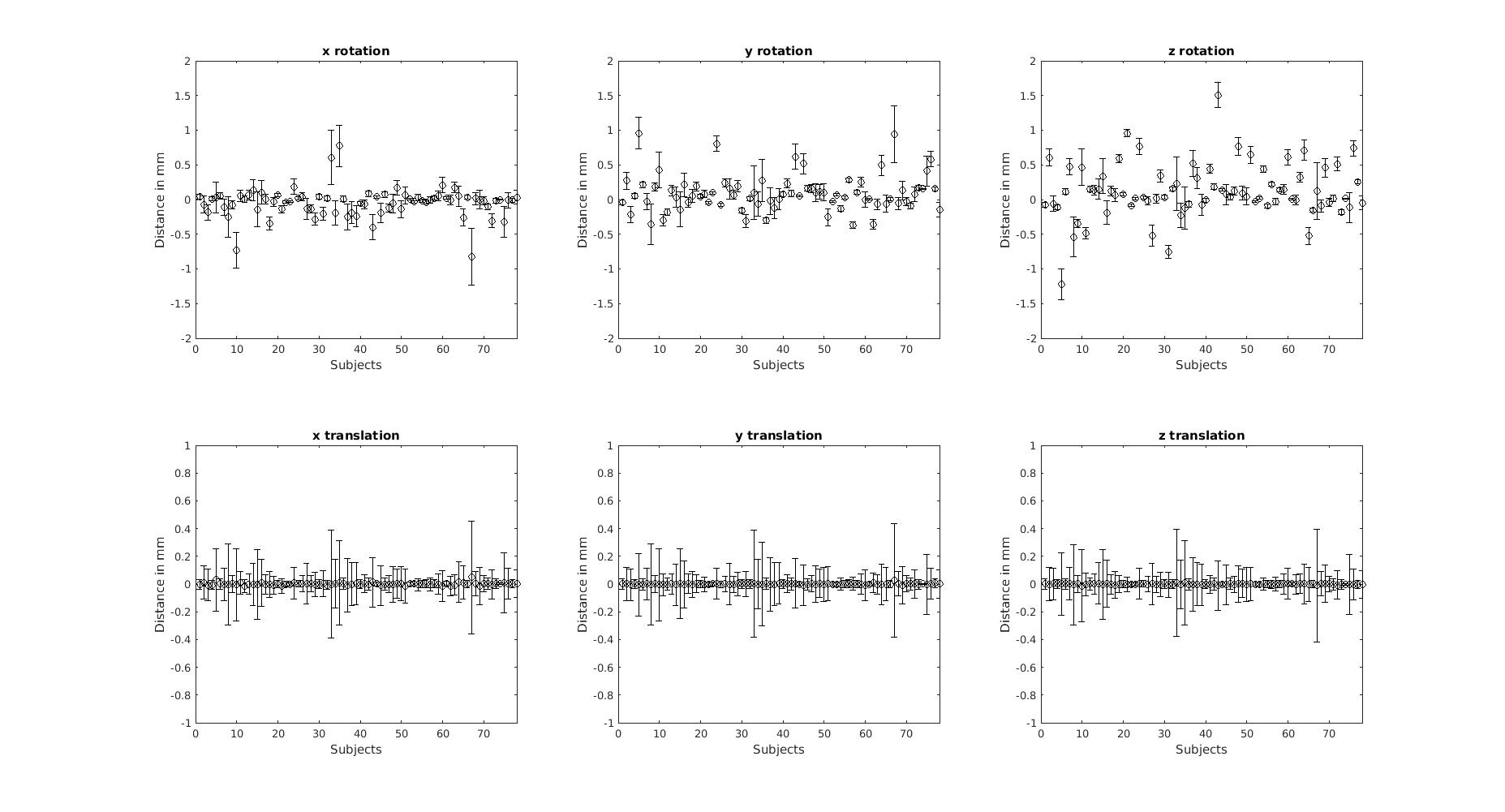
**

Fig. S5: Averages and standard deviations of the 6 motion correction parameters applied to each subject.

**Effects of motion: DVARS and Framewise Displacement analysis**

|  | Patients | Controls |
| --- | --- | --- |
| Framewise Displacement |  |  |
| Mean | 0.3833±0.05 | 0.3245±0.29 |
| Min. | 0.1081 | 0.0683 |
| Max. | 1.6864 | 0.8082 |
| DVARS |  |  |
| Mean | 22.82±7.24 | 18.779±4.67 |
| Min. | 13.8 | 11.45 |
| Max. | 47.61 | 59.16 |

TABLE S1: Analysis of volumes affected by residual motion effects by two framewise indices of data quality, Framewise Displacement (FD) and DVARS. Outlier volumes were identified by FSL using the function fsl_motion_outliers for the two metrics. No significant difference between patient and control groups was detected in the number of volumes nor in the percentage of scan-time affected, supporting the idea that schizophrenia patients included in this study do not show increased motion compared to controls.


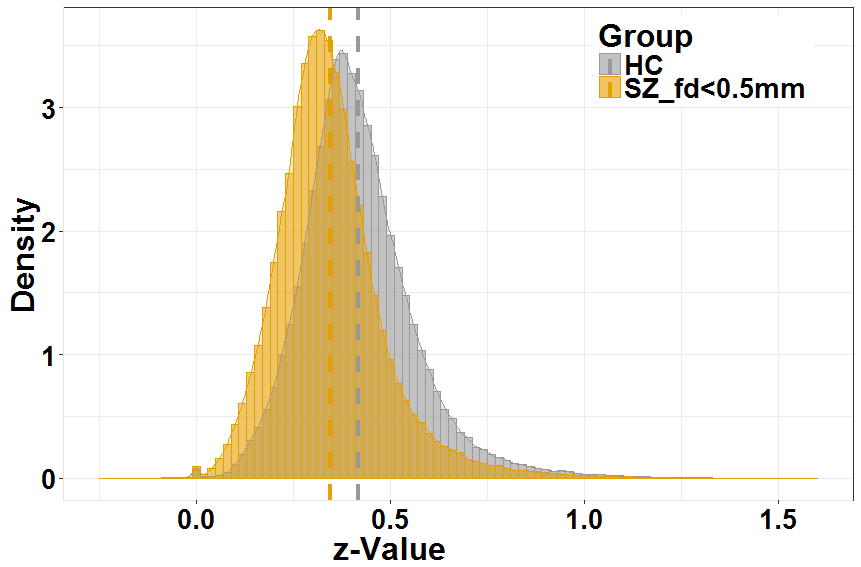


Fig. S6: Histogram of z.transformed edge weights after removing subjects with FD greater than 0.5mm as measured by Framewise Displacement. A total of 61 patients were included in this analysis. Even upon removal of subjects with movement above this very stringent threshold, the edge strength distribution of patients remains weaker than in healthy controls, and virtually identical to that shown in Fig. 1. This corroborates the idea that differences between patients and controls are not driven by a few subjects exhibiting increased motion.

## Anatomical/functional identification of the 15 biggest modules of each population

|  | Control | Patient |
| --- | --- | --- |
| 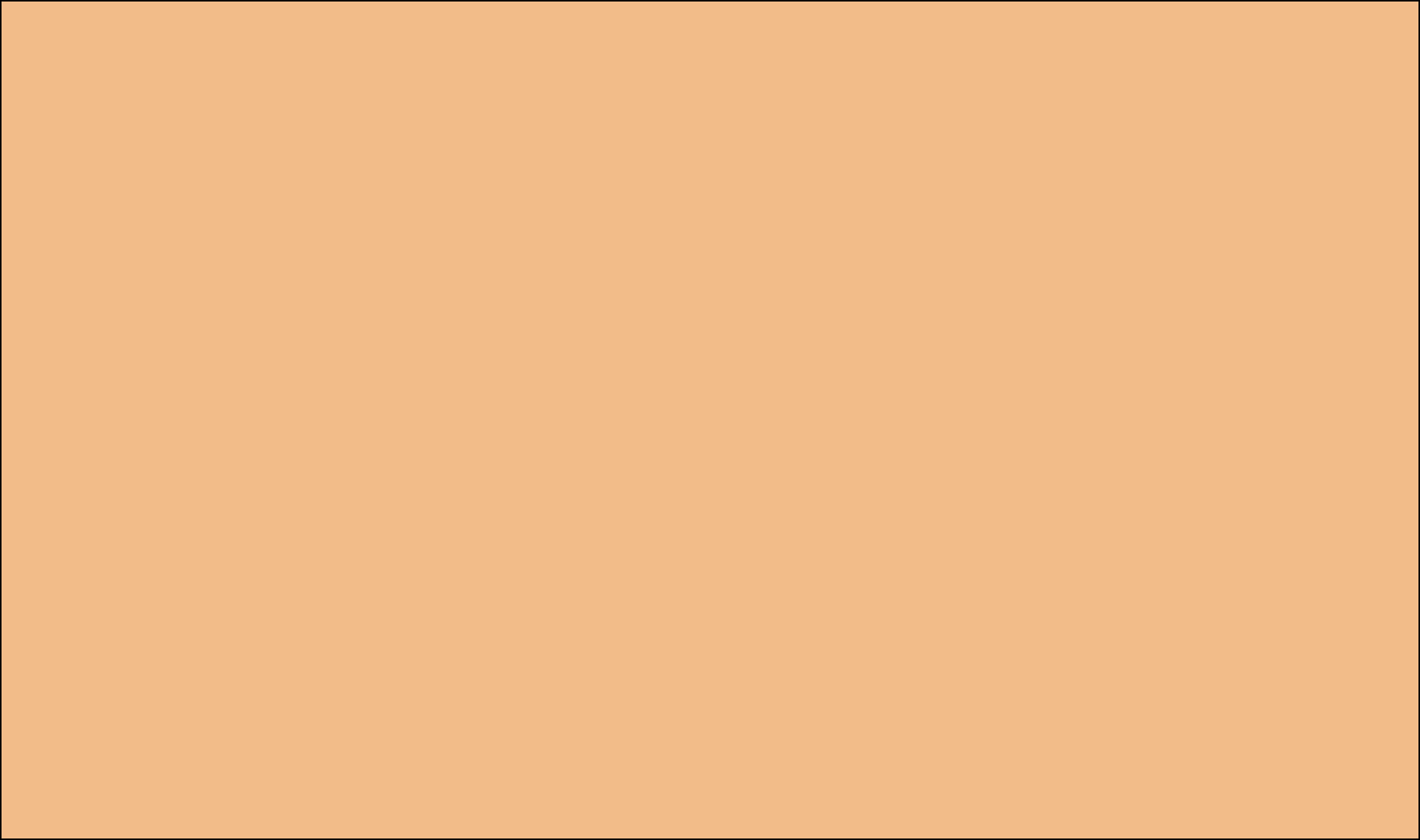 | Module1: Visual | Module1: Visual (primary) |
| 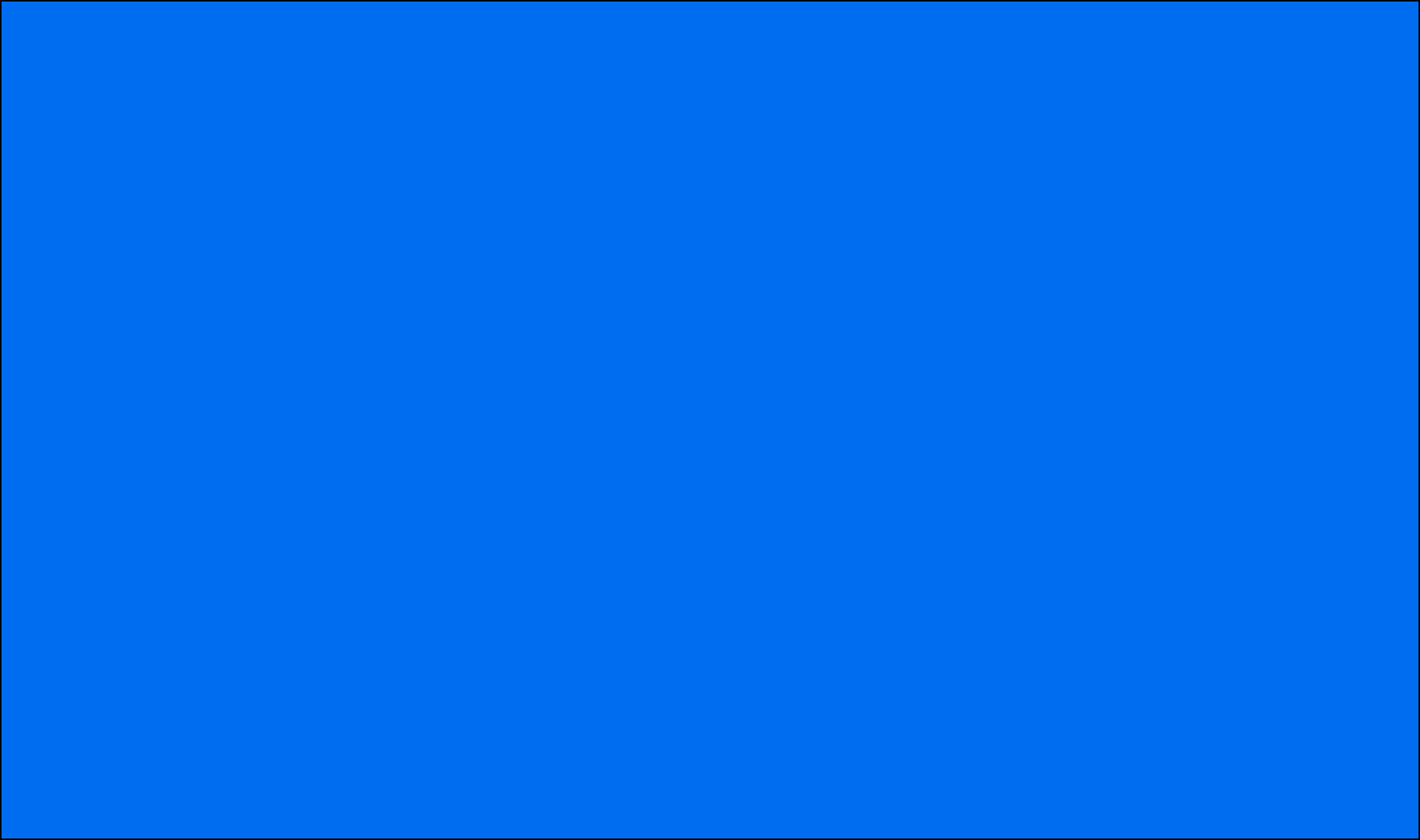 | Module2: Temporal-Somatosensory | Module2: Visuo-parietal |
| 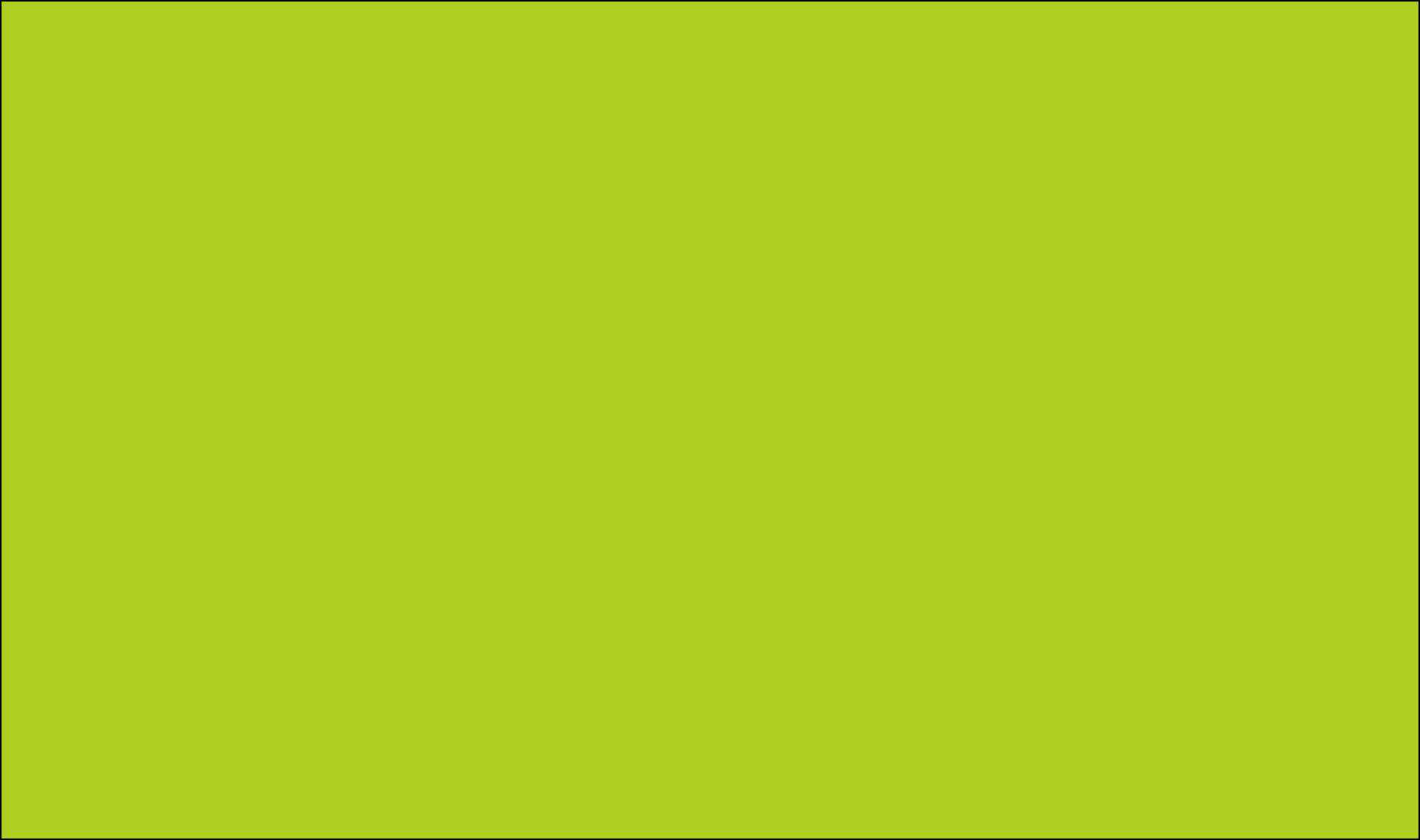 | Module3: Frontal (DMN) | Module3: Somatosensory (lateral) |
| 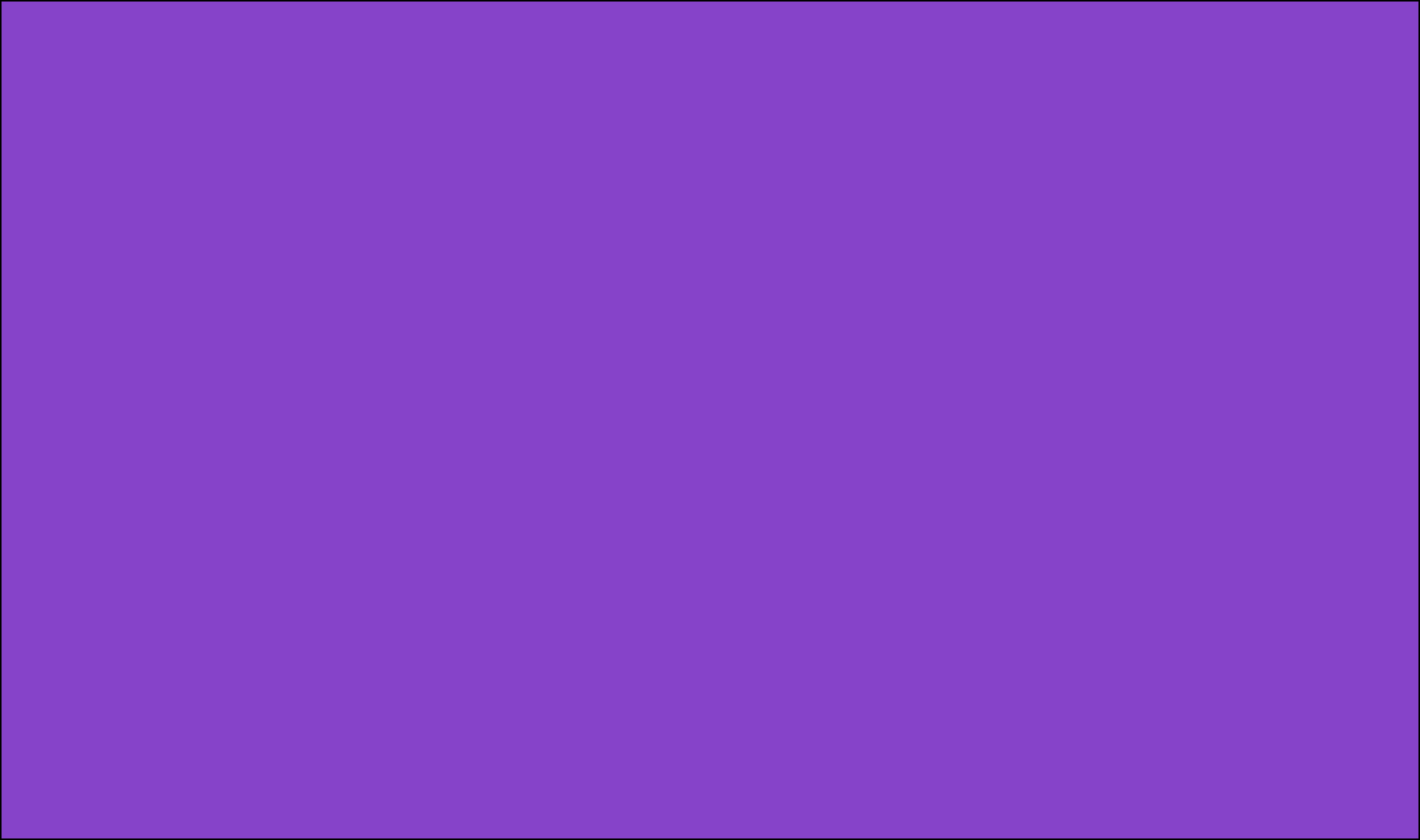 | Module4: Frontal (attention network) | Module4: Frontal (DMN) |
| 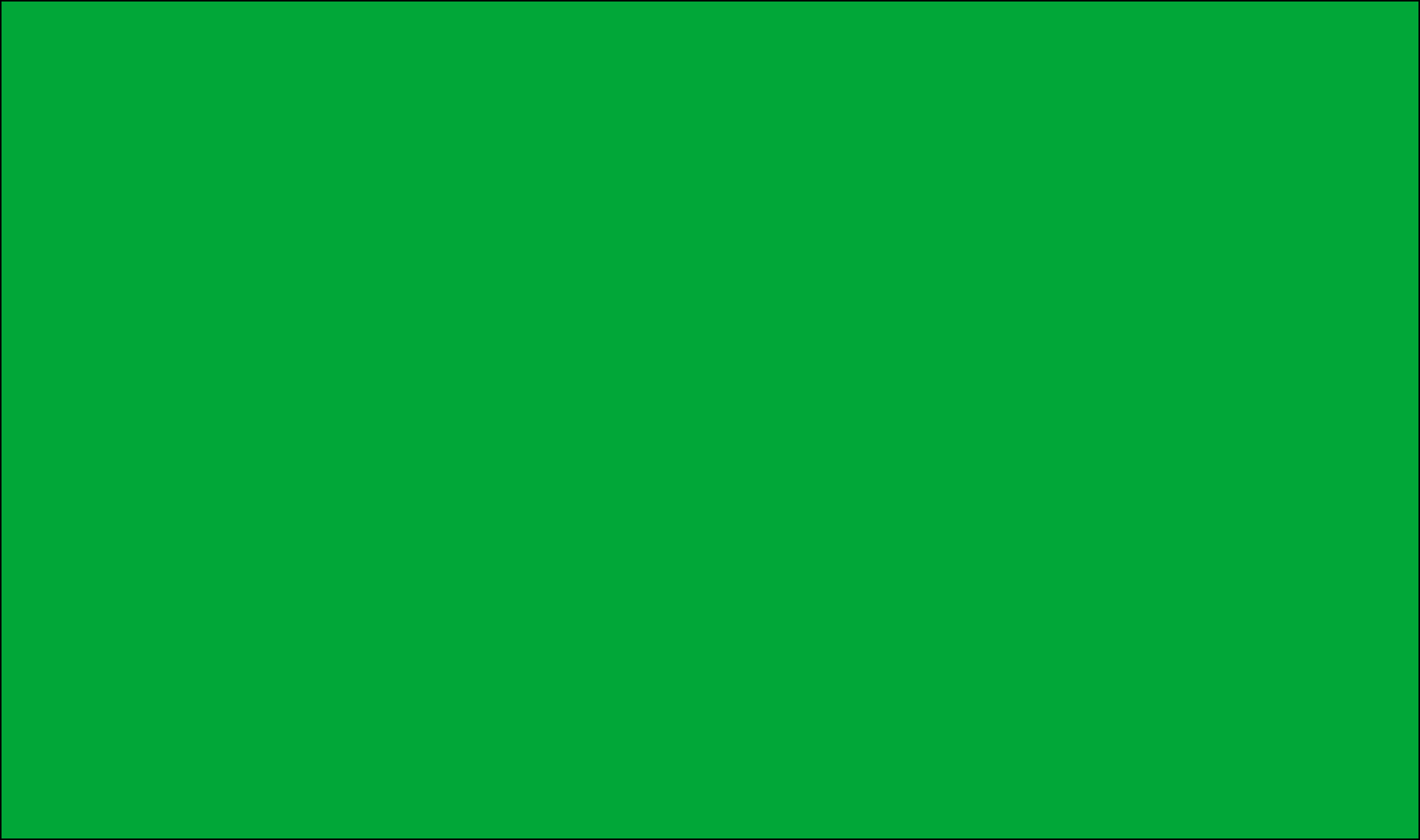 | Module5: Temporal (inferior) | Module5: Temporal superior (insula-rolandic oper.) |
| 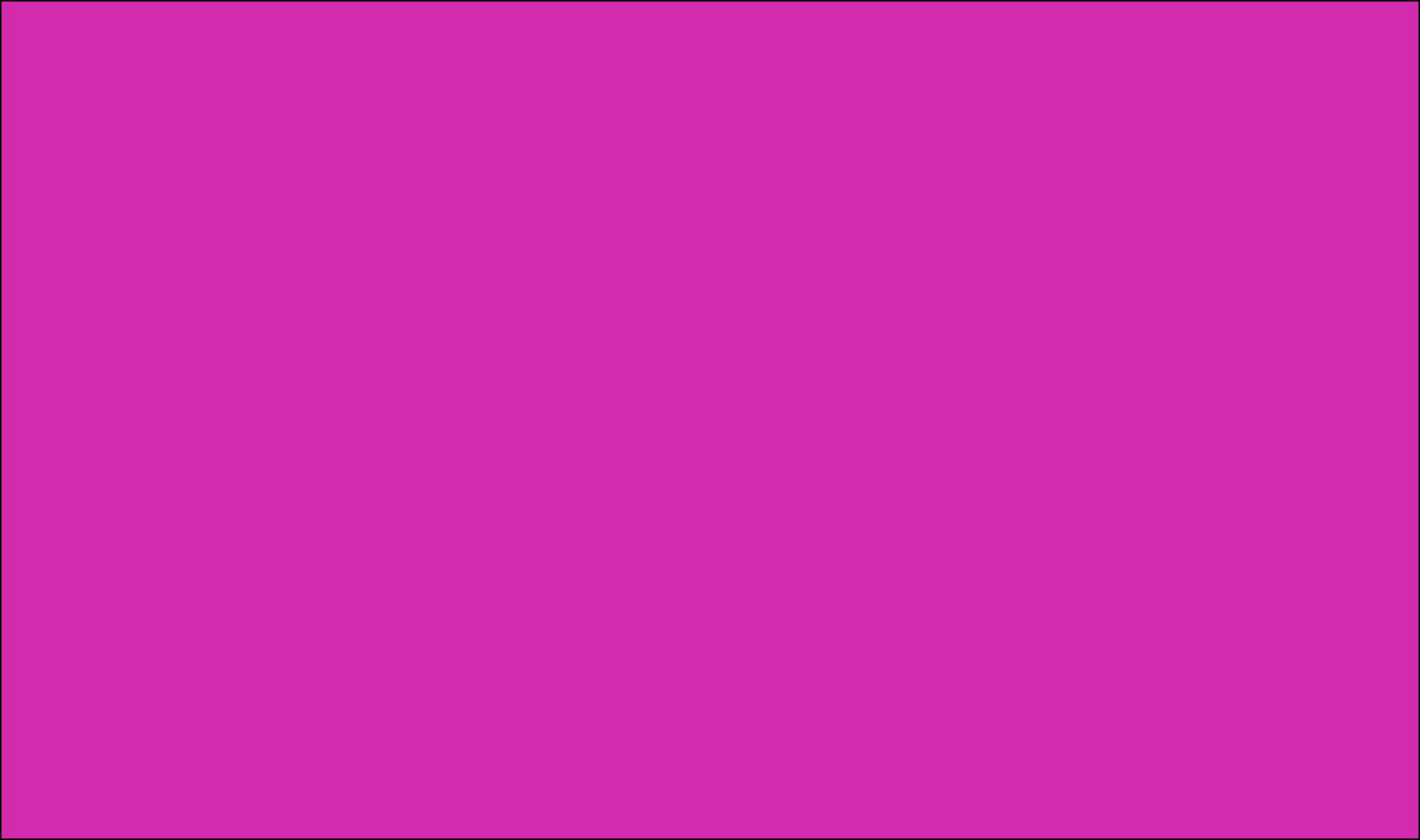 | Module6: Frontal | Module6: Somatosensory |
| 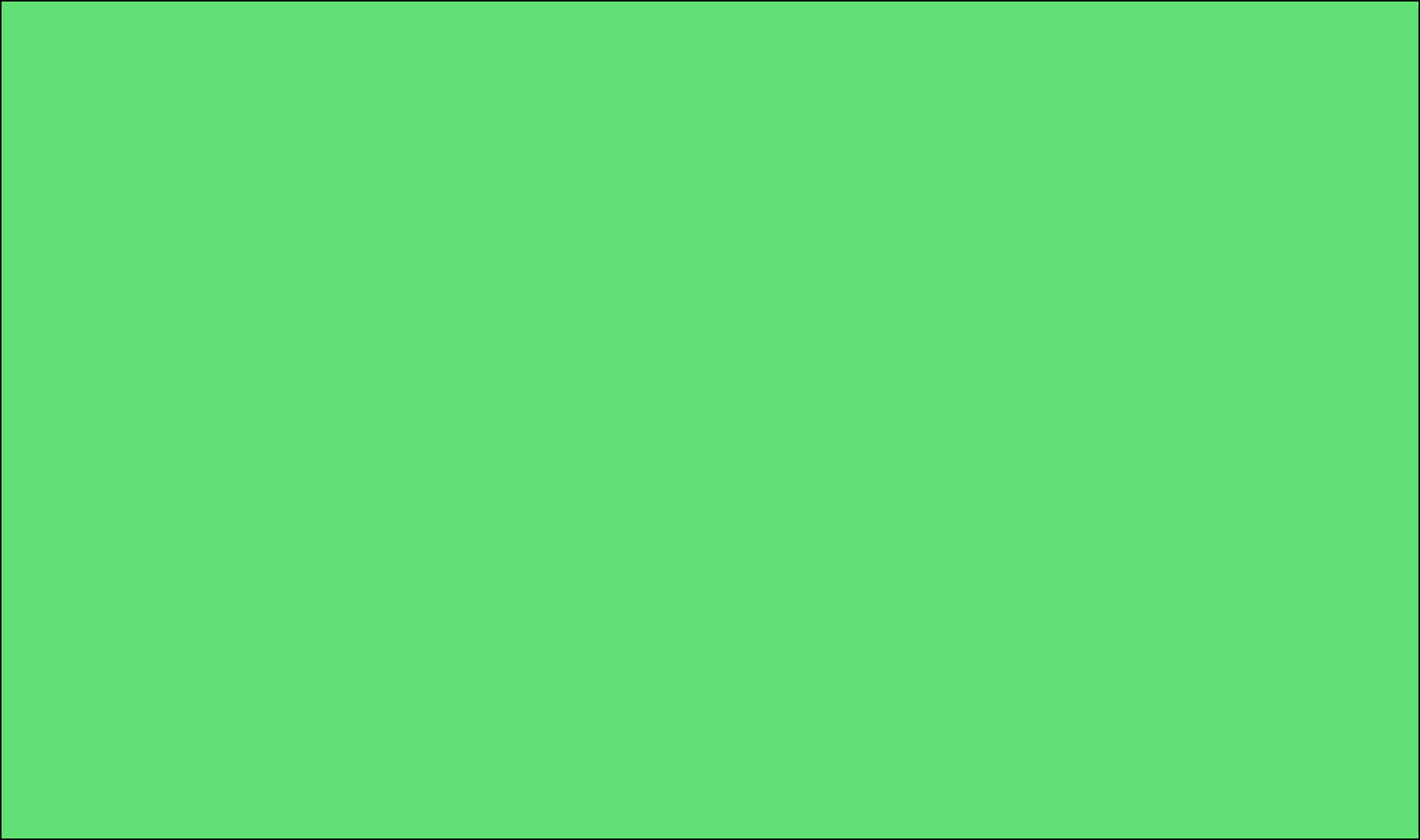 | Module7: Temporal (middle) | Module7: Visual (ventral) |
| 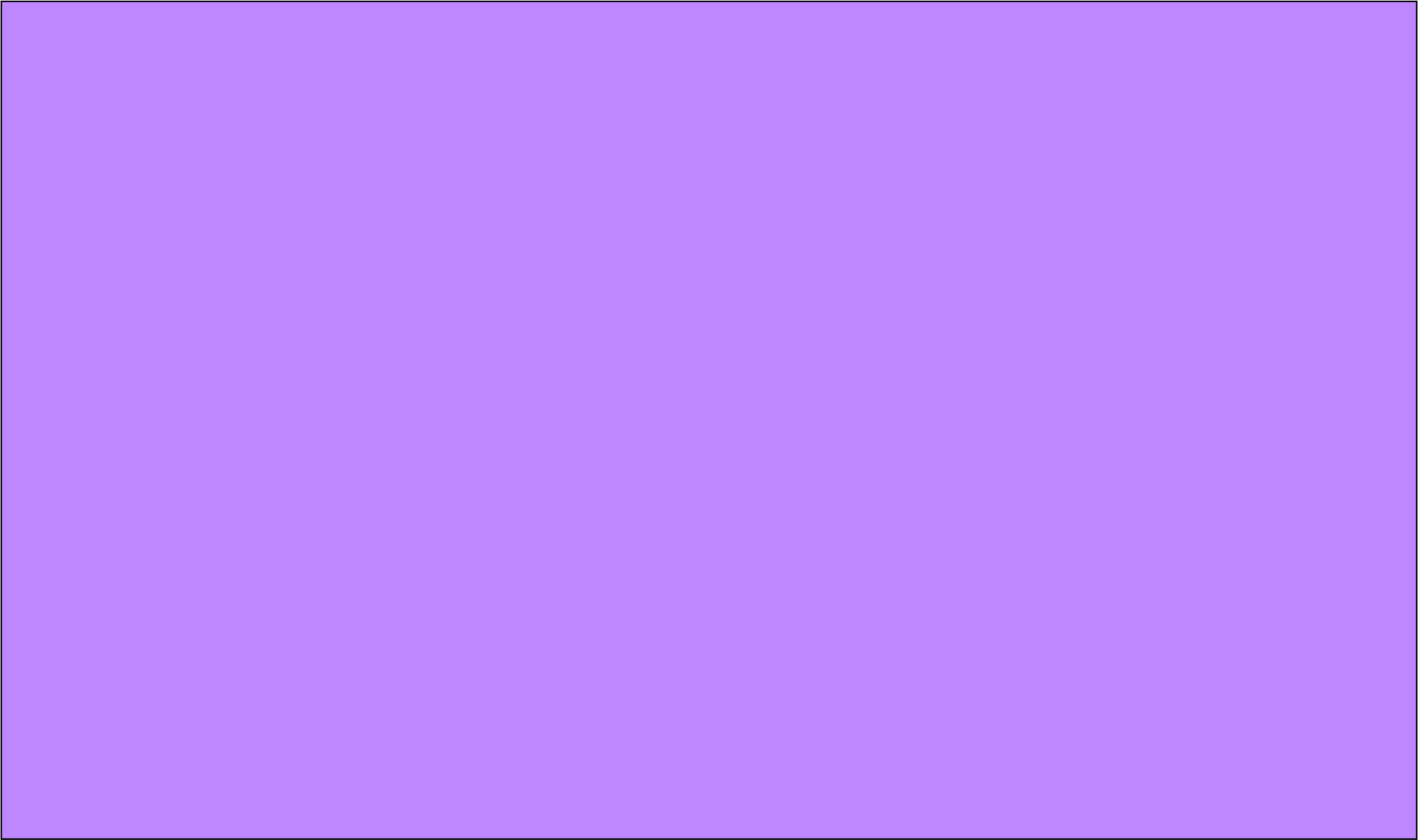 | Module8: Basal | Module8: Temporal (inferior) |
| 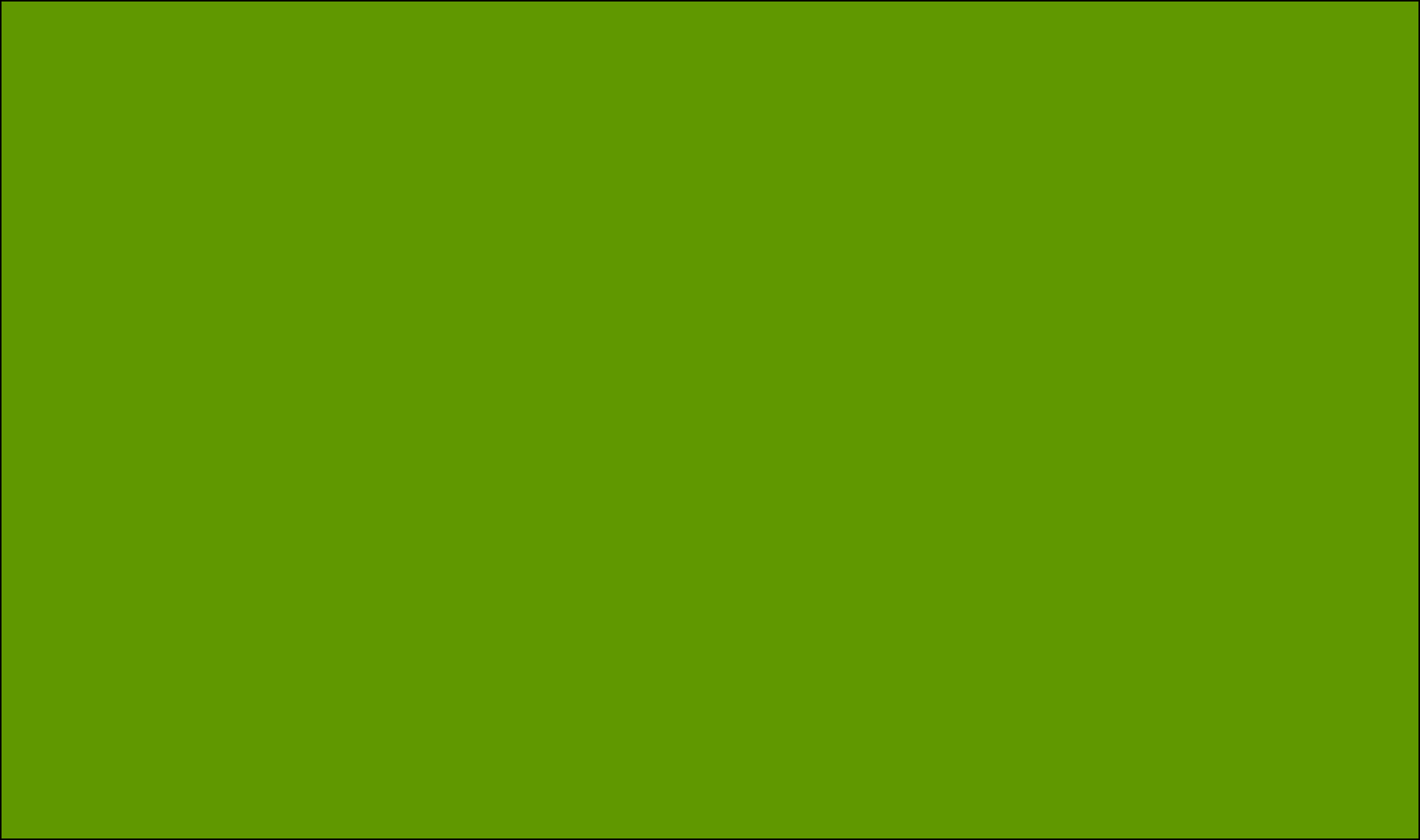 | Module9: Parietal | Module9: Frontal |
| 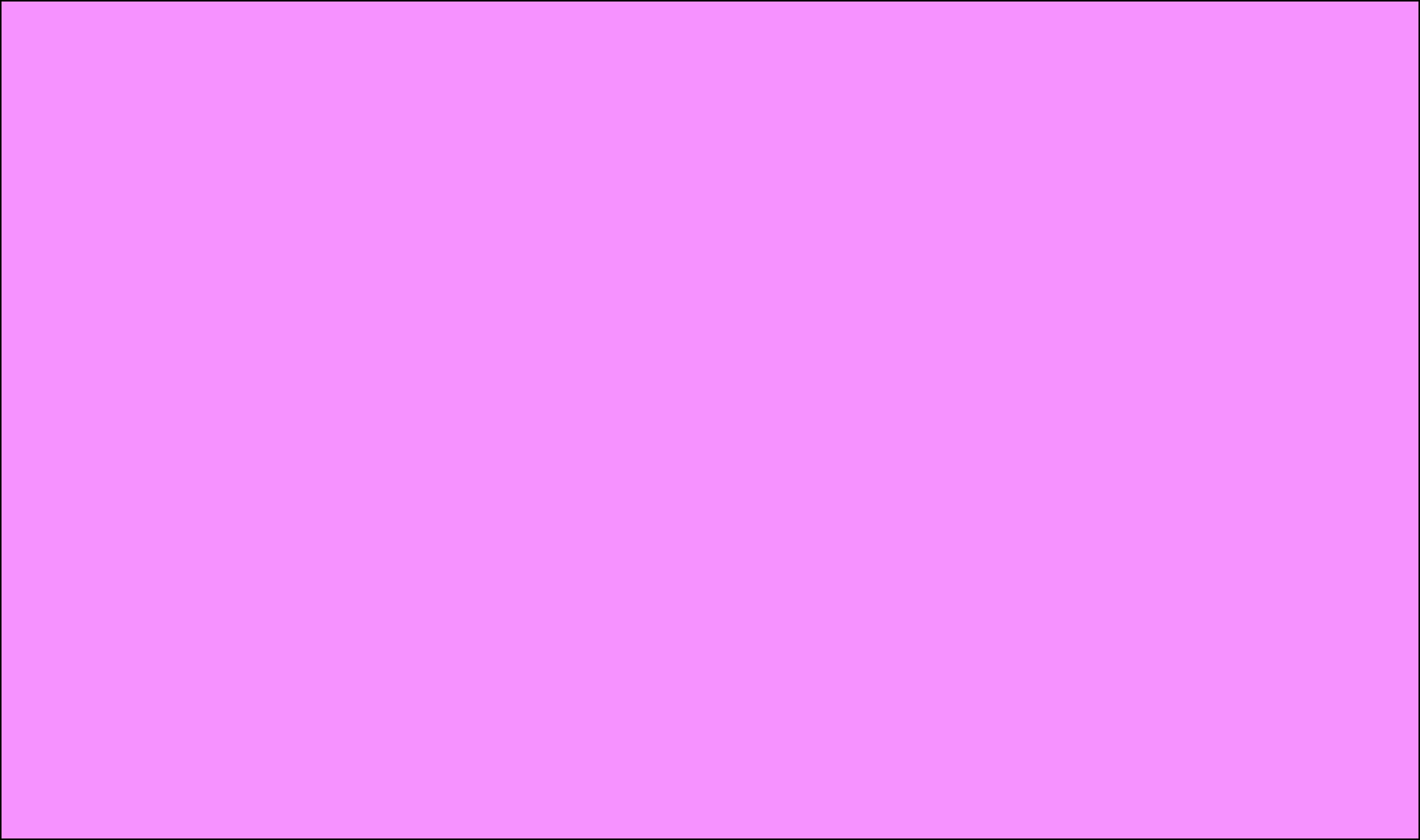 | Module10: Fronto orbital | Module10: Fronto orbital |
| 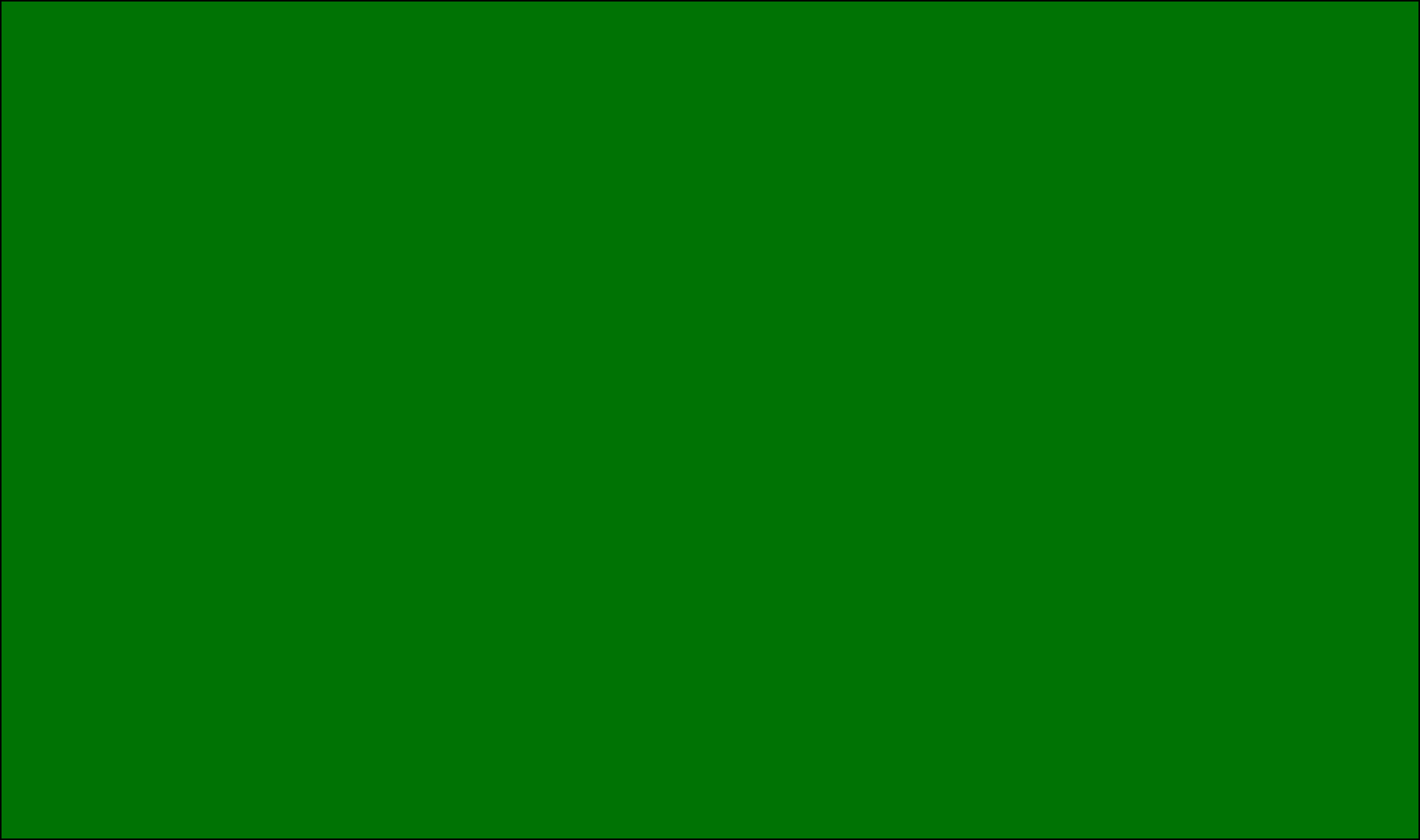 | Module11: Cingulum (executive network) | Module11: Postcentral/ Precuneus |
| 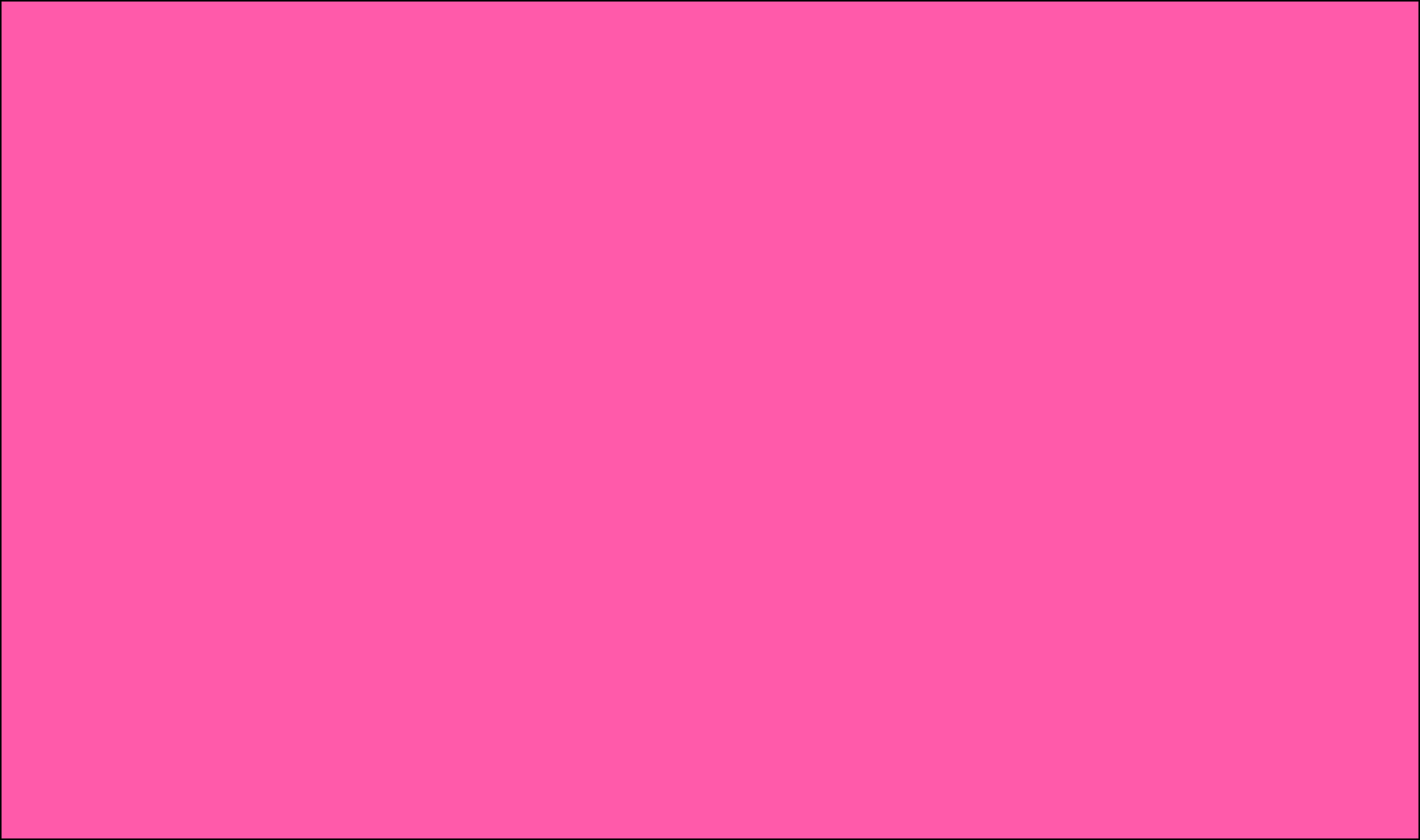 | Module12: Postcentral-precuneus | Module12: Basal |
| 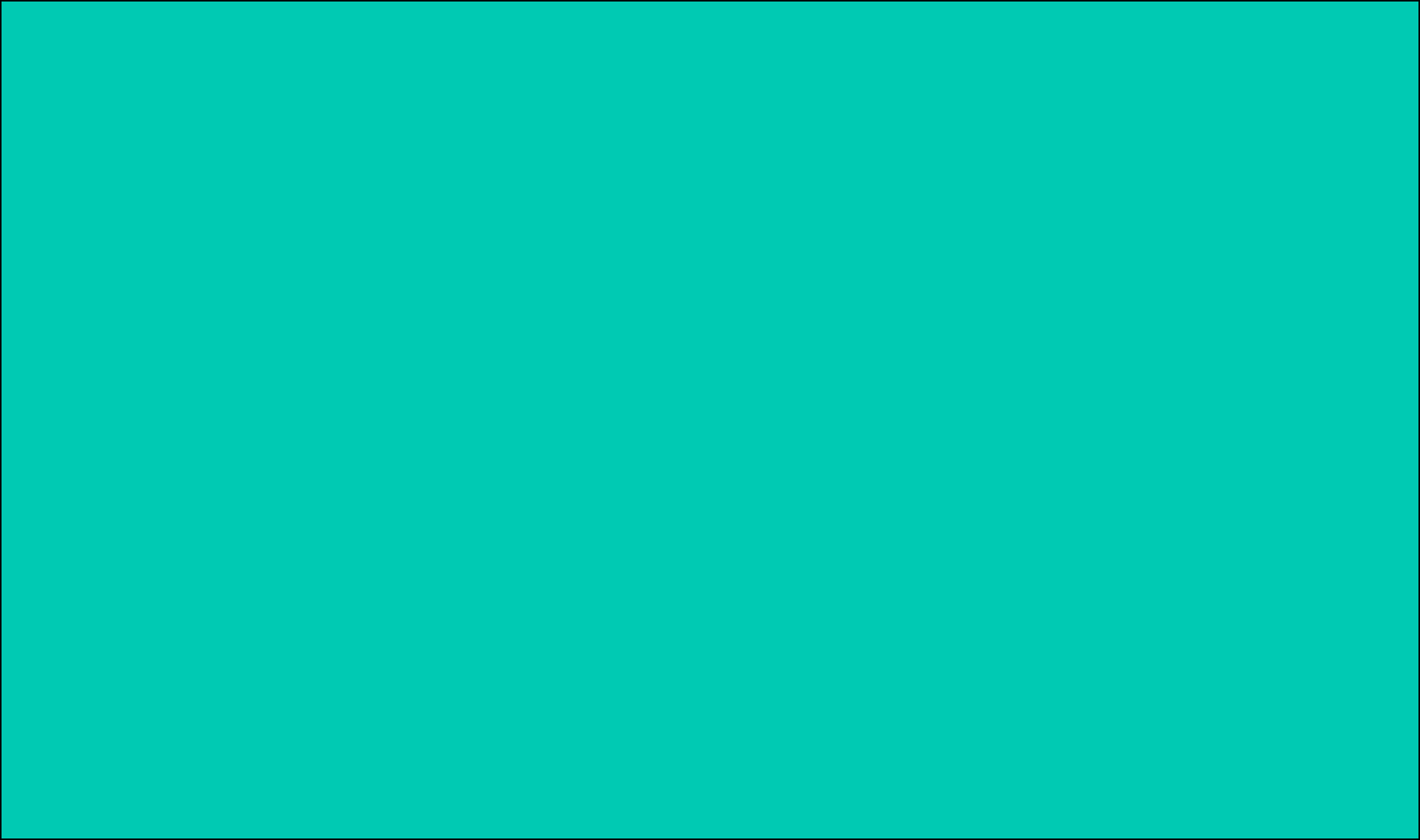 | Module13: Angular | Module13: Frontal (attention network) |
| 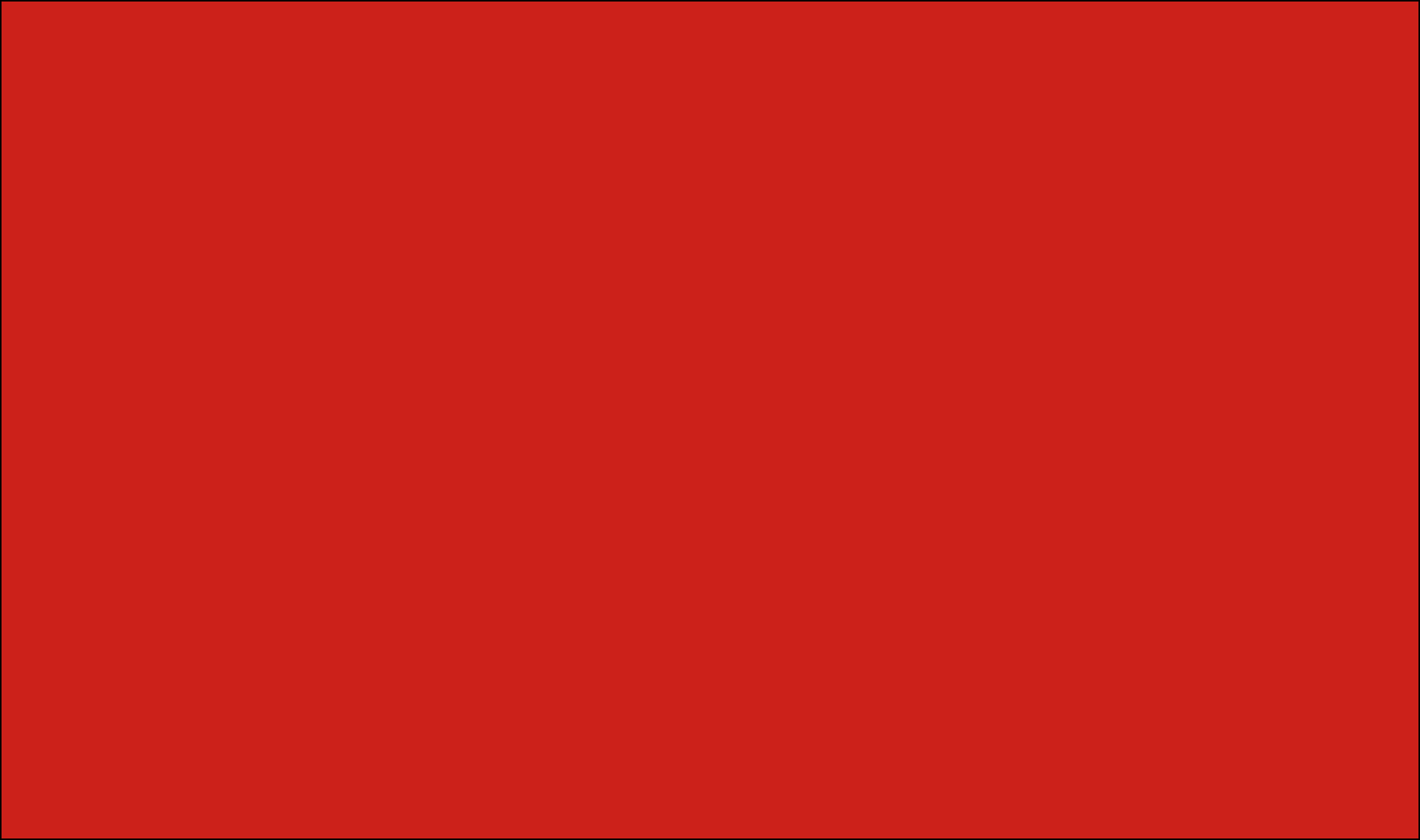 | Module14: Middle Fronto-orbital | Module14: Supramarginal/ Temporal (middle) |
| 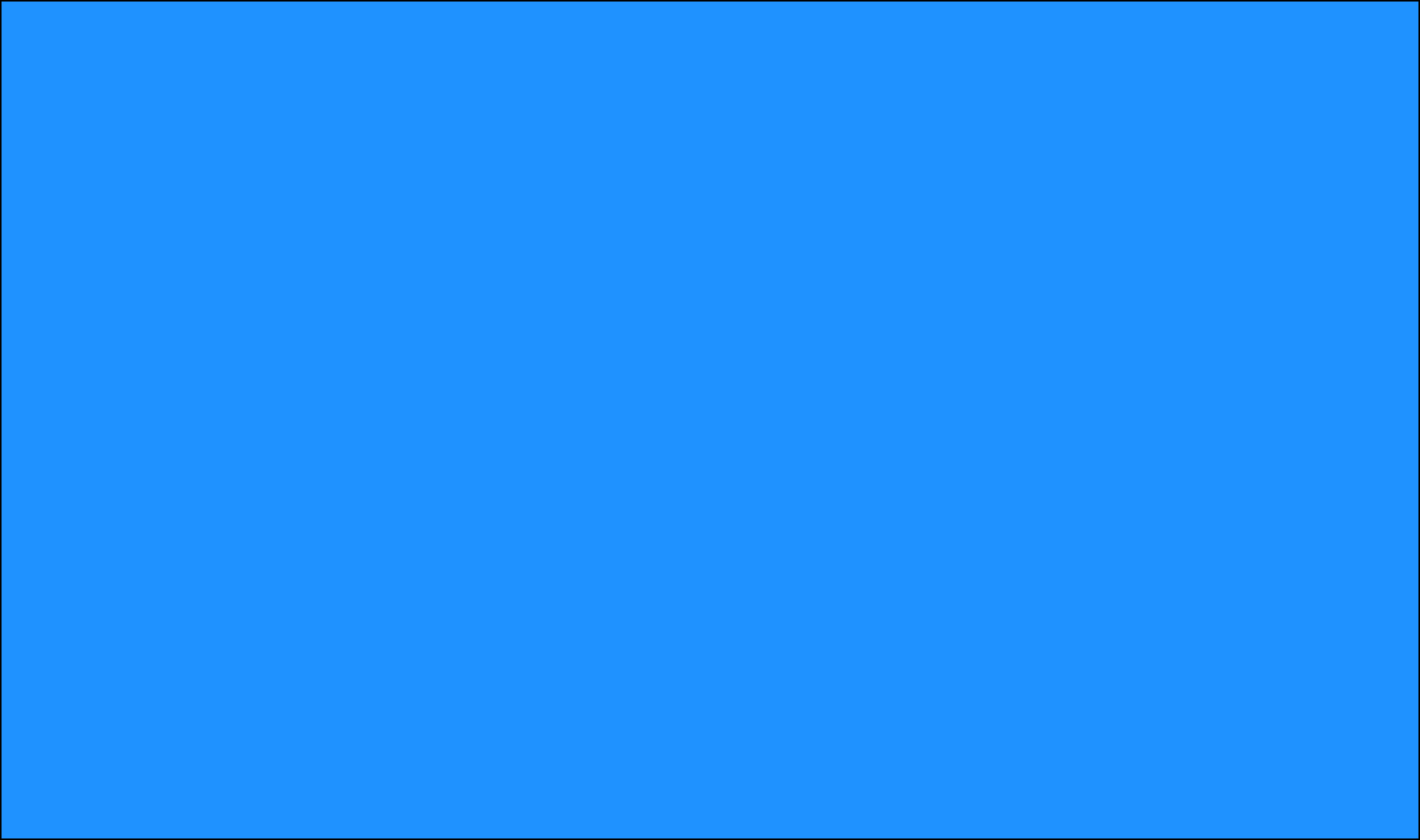 | Module15: Amygdala-hyppocampal | Module15: Cingulum (executive network) |

TABLE S2

**Statistical comparison of SZ with FD<0.5 against HC**

**Framewise Displacement**

**
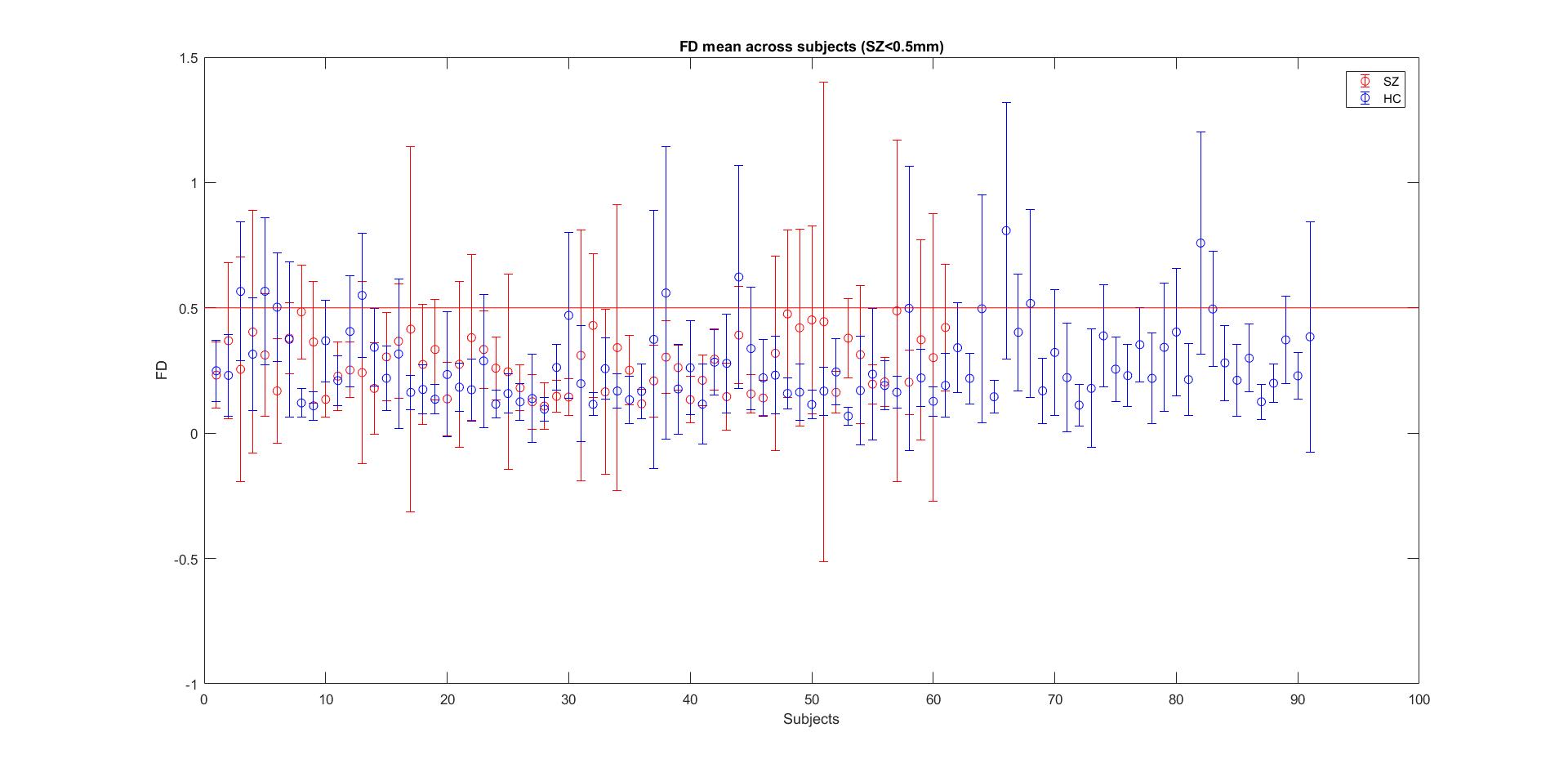
**

Fig. S7a: Comparison of Framewise Displacement of the subgroup of schizophrenia subjects against healthy controls; no statistically significant difference is observed between these two groups.

**Mean FD :** HC=0.275±0.15**;** SZ=0.279±0.1

**Statistical comparison:** Ttest: t=-0.179**;** p=0.858

**DVARS**

**
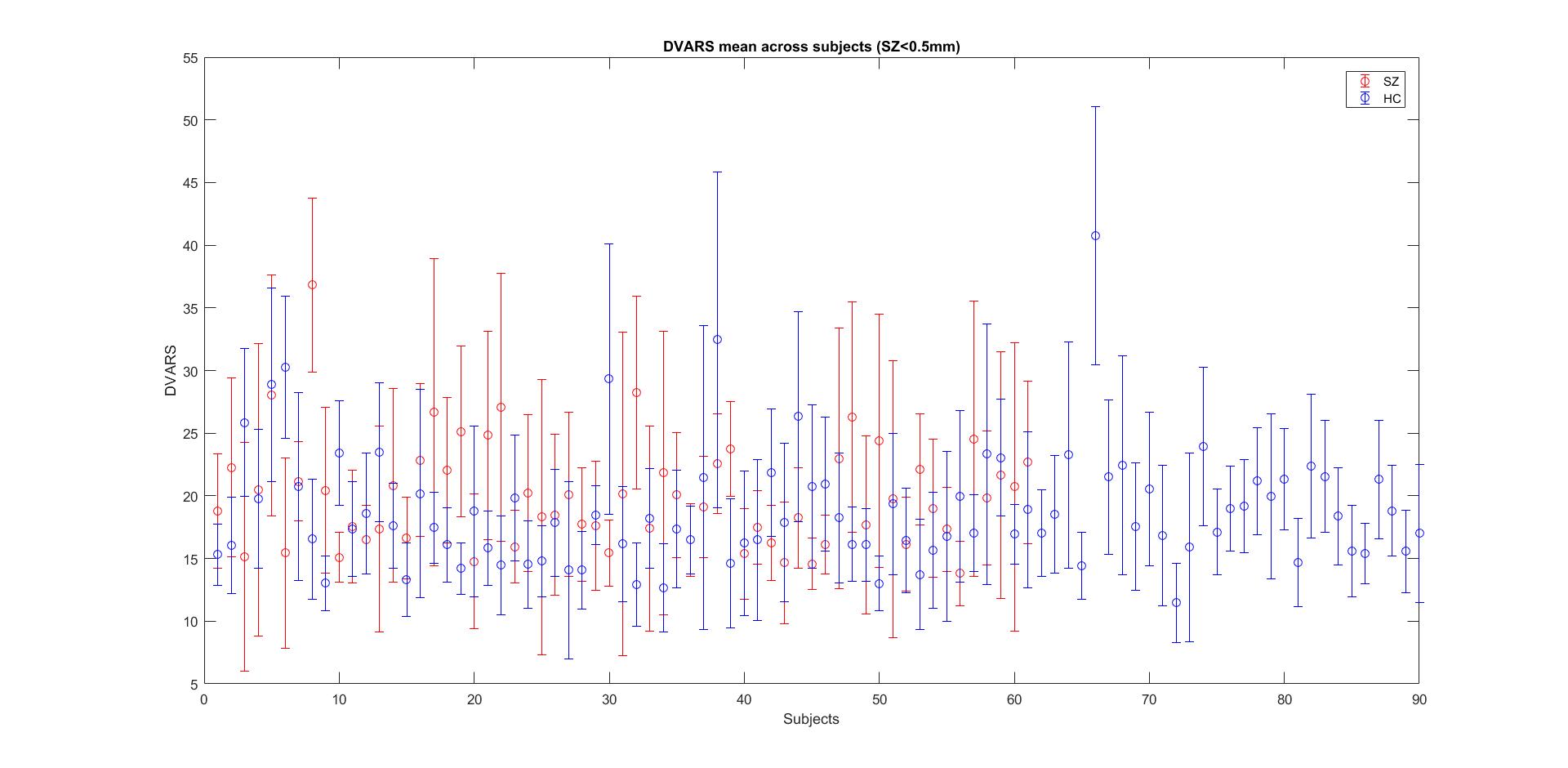
**

Fig. S7b: Comparison of DVARS of the subgroup of schizophrenia subjects against healthy controls; no statistically significant difference is observed between these two groups.

**Mean DVARS:** HC=18.78±4.67**;** SZ=20.01±4.27

**Statistical comparison:** Ttest: t=-1.653; p=0.100

**SZ with FD <0.5 mm (Power et al., 2012) – 61 Subjects**


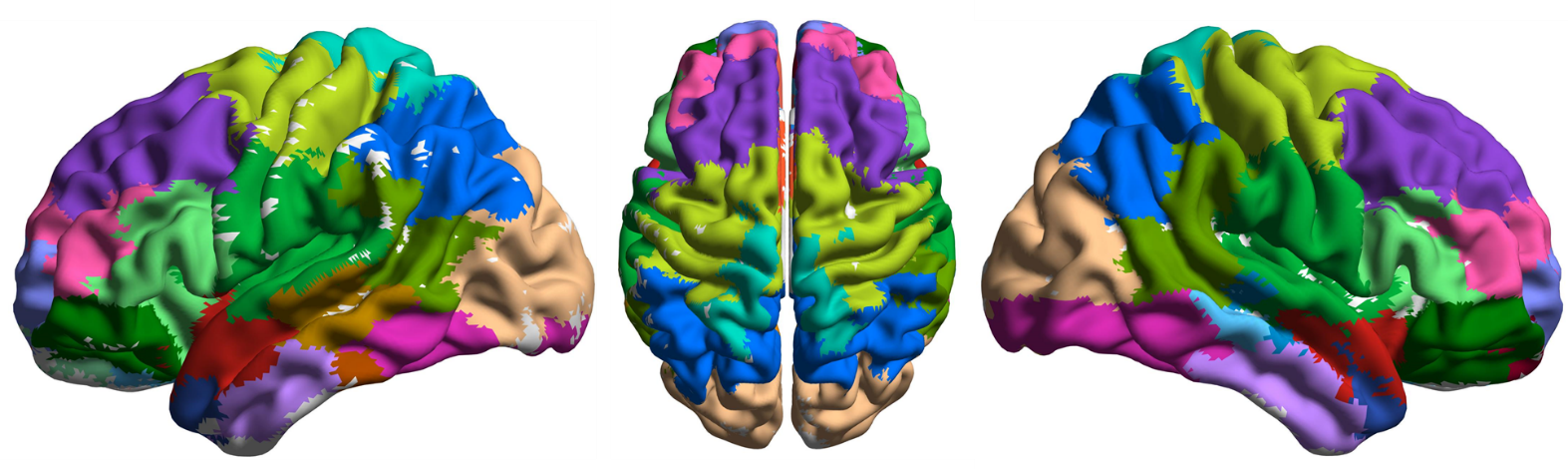


Fig. S8: Modularity analysis by Asymptotical Surprise in a subgroup of 61 patients with FD < 0.5mm. Fragmentation of sensory cortices is clearly detectable in this subgroup of patients selected by a strict motion-control criterion. This supports the idea that modular reorganization is not the result of increased motion in the patient group, and is not driven by a subgroup of subjects characterized by increased motion.
